# Impact of an interactive web tool on patients’ intention to receive COVID-19 vaccination: a before-and-after impact study among patients with chronic conditions in France

**DOI:** 10.1186/s12911-021-01594-8

**Published:** 2021-07-31

**Authors:** Viet-Thi Tran, Stéphanie Sidorkiewicz, Clarisse Péan, Philippe Ravaud

**Affiliations:** 1 CRESS, INSERM, INRA, Université de Paris, 75004 Paris, France; 2 Centre d’Épidémiologie Clinique, Hôpital Hôtel-Dieu, AP-HP, 75004 Paris, France; 3 Département de medecine generale, Université de Paris, 75014 Paris, France; 4 ASSOMAST French Patients’ Organization, Paris, France; 5 Department of Epidemiology, Columbia University Mailman School of Public Health, 22 W 168th St, New York, NY USA

**Keywords:** COVID-19, Vaccine, Decision aids

## Abstract

**Objectives:**

In France, about 30% of the population refuses COVID-19 vaccination outright, and 9 to 40% are hesitant. We developed and evaluated an interactive web tool providing transparent and reliable information on the benefits and risks of COVID-19 vaccination.

**Methods:**

The most recent scientific data at the time of the study were implemented into an interactive web tool offering individualized information on the risks of COVID-19 infection-related events versus vaccination-related serious adverse events. The tool was evaluated during a before-and-after impact study nested in ComPaRe, a French e-cohort of adult patients with chronic conditions. Primary outcome was the proportion of patients intending to receive vaccination after using the tool, among those not intending to receive it at baseline.

**Results:**

Between January 8 and 14, 2021, we enrolled 3152 patients in the study [mean age 55.2 (SD: 16.9), 52.9% women and 63% with ≥ 2 chronic conditions]. Before consulting the tool, 961 (30.5%) refused to be vaccinated until further data on efficacy/safety was obtained and 239 (7.5%) outright refused vaccination. Among these 1200 patients, 96 (8.0%, number needed to treat: 12.5) changed their mind after consulting the tool and would subsequently accept vaccination.

**Conclusions:**

Our interactive web tool represents a scalable method to help increase the intent to receive COVID-19 vaccination among patients with chronic conditions and address vaccine hesitancy. Since April 2021, our tool has been embedded on the official webpage of the French Government for COVID-19 information.

**Supplementary Information:**

The online version contains supplementary material available at 10.1186/s12911-021-01594-8.

## Introduction

Global vaccination against SARS-CoV-2 has raised hopes of putting an end to the current global COVID-19 pandemic. Yet, vaccine hesitancy, which is defined as a “delay in acceptance or refusal of vaccination despite availability of vaccination services” could compromise this endeavor [1]. Vaccine hesitancy has been an issue in France long before the COVID-19 pandemic, and the country ranked among the ten least confident countries regarding vaccine safety in a survey conducted across 67 countries in 2016 [2]. The vaccination campaign for COVID-19 in France is not spared from vaccine hesitancy, and a recent study showed that 30% of people in the working-age population would refuse COVID-19 vaccination outright, and 9 to 40% of them are hesitant [3]. Common reasons for vaccine refusal and hesitancy include concerns about the vaccine safety and effectiveness, or a need for more information, especially given the fast development of the vaccines [4]. More recently, concerns on the safety of two Adenovirus based vaccines (from AstraZeneca and Johnson & Johnson) may deter people who are already hesitant and give room for the spread of misinformation [5].

In this context, decision aids based on transparent and accurate scientific information could help physicians and patients better estimate the benefits and risks of vaccination, and make informed choices regarding vaccination.

In this study, we aimed at assessing the impact of an interactive web tool on COVID-19 vaccination's benefits and risks on the intention to receive vaccination among individuals with chronic conditions, at a very early stage of the vaccination campaign in France.

## Methods

Our study comprised two stages: first, the development of an interactive web tool offering individualized information on the benefits and risks of COVID-19 vaccination, and second, the assessment of its impact on intention to receive vaccination during a before-and-after impact study.

### Development of the tool

**Fig. 1:**
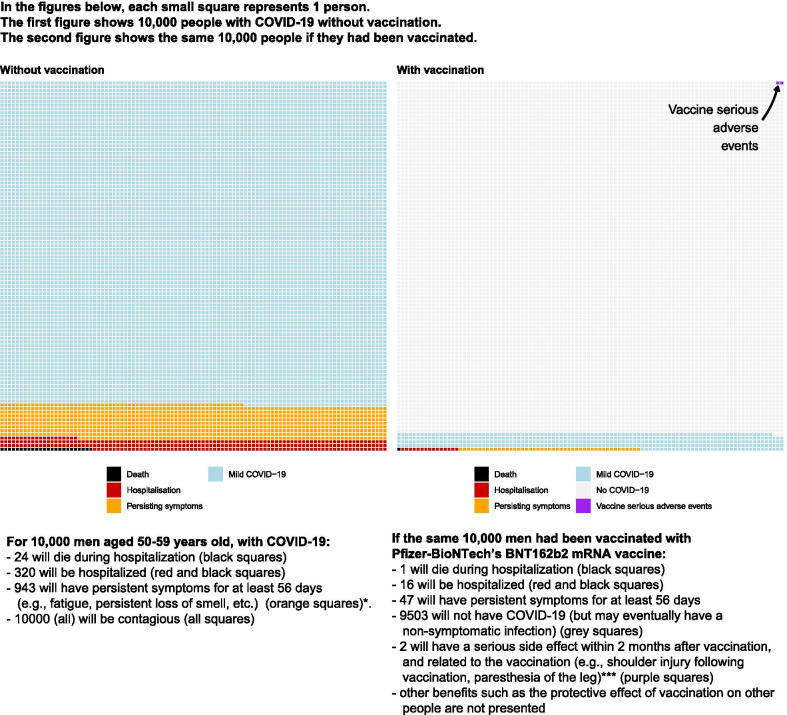
Example of the tool’s output for a man, aged 50–59 years old, and using Pfizer-BioNTech’s BNT162b2 mRNA vaccine

From December, 26th 2020 to January, 6th 2021, two epidemiologists and one primary care physician involved in the vaccination campaign (VTT, SS, PR) developed an interactive web tool that aimed at offering individualized information on the risks of death, hospitalization, symptom persistence at 2 months, in case of COVID-19 infection, with and without vaccination, and on the risks vaccination-related serious adverse events. The tool was based on the most recent scientific data available at that time and focused on evidence-based methods to counter antivaccination attitudes [6–10]. Its design was inspired by existing decision aids and its content followed the recommendations from the International Patient Decision Aid Standards (IPDAS) [11, 12]. Output of the tool could be personalized according to gender, age, and types of vaccine and used 10 000-person pictographs to illustrate the absolute risk reduction and the serious adverse effects associated with vaccination (Additional file 1). Three patients aged 33 to 78 years old pilot-tested and helped improve the tool’s clarity and wording before its release at https://cress-umr1153.fr/covid_vaccines/ (Fig. 1).

### Assessment of the impact of the tool on patients’ intention to receive COVID-19 vaccination

We evaluated the impact of the tool on patients’ intention to receive COVID-19 vaccination during a before-and-after impact study nested in ComPaRe (Community of Patients for Research), a nationwide e-cohort of patients with chronic conditions, in France (www.compare.aphp.fr) [13].

#### Participants

Participants were adult patients (> 18 years old) reporting having at least one chronic condition, defined as any medical condition requiring healthcare for at least 6 months. Participants who reported a previous SARS-CoV2 infection (laboratory confirmed or not) were excluded from the study because, at the time of the study, they were not yet eligible for vaccination. Participants provided written informed consent before participation. ComPaRe was approved by the Institutional Review Board of Hôtel-Dieu Hospital, Paris (IRB: 0008367). Participants were not exposed to any form of harm as a result of their participation in this study.

#### Data collected and outcomes

Before consulting the tool, participants were asked to select their intention to receive COVID-19 vaccination from four response options (“Yes, with any vaccine,” “Yes, but not with all vaccines,” “No, I prefer to wait for more vaccine efficacy/safety data,” and “No, I don’t want to be vaccinated at all”). Then, they were presented the web interactive tool, embedded in the study questionnaire, and invited to test it. Finally, after consulting the tool, participants were immediately asked to reassess their intention to receive COVID-19 vaccination, using the same four response options as before.

The primary outcome was the proportion of patients intending to be vaccinated after using the tool, among those not intending so at baseline. Secondary outcomes included respondents’ perception of the tool's usefulness and of the importance of vaccination at the individual and population levels, assessed by rating scales ranging from 0 (not useful/important) to 100 (extremely useful/important).

Demographic and clinical characteristics of participants (i.e., age, sex, educational level, comorbidities, number of people living with the participant) were collected as part of the ComPaRe baseline data collection.

#### Analysis

All analyses used a weighted dataset obtained by calibration on margins with weights for age, gender, and educational level derived from national census data describing the French population of patients with chronic conditions [14]. We performed a post-hoc multivariable logistic regression, accounting for the weighted data, to investigate the association between change of mind and patients’ characteristics. Variables included in the model were sex, age (as a continuous variable), high educational level, household with > 1 adult (including the patient), household with ≥ 1 children, living with a person over 65 years old, living with a person having a chronic condition and responses to the “before” assessment. Analyses were performed on complete cases only. *P* < 0.05 was considered statistically significant.

All statistical analyses involved the use of R v3.6.3 (http://www.r-project.org, R Foundation).

## Results

### Participants

**Fig. 2:**
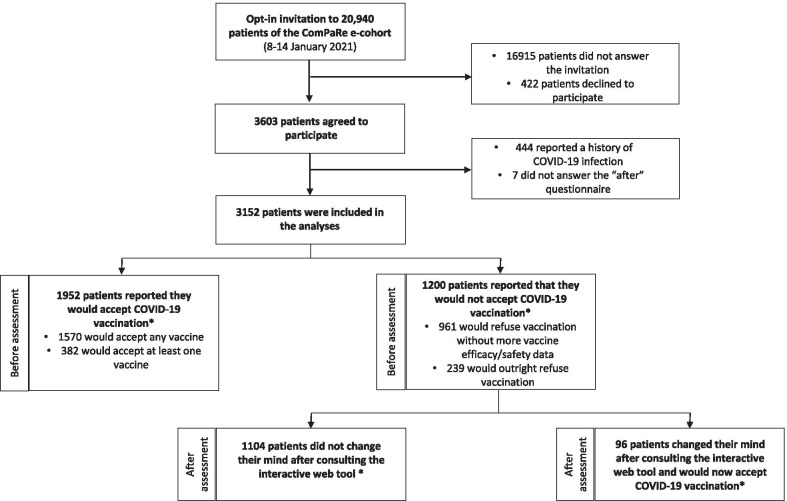
Study flow chart *numbers are presented after weighting

From January 8 to 14, 2021, we sent an opt-in invitation to 20,940 members of the ComPaRe e-cohort and 3603 patients volunteered to participate (422 declined to participate). Finally, 3152 patients were included in the analyses (451 were further excluded because they reported a history of COVID-19 infection [n = 444], or did not complete the “after” questionnaire [n = 7]) (Fig. 2). In the weighted dataset, their mean age was 55.2 (SD: 16.9) and 1666 (52.9%) were women. About 63% of patients had multimorbidity (the mean number of conditions was 2.8 (SD: 2.7). Patients’ conditions included, among others, high blood pressure (n = 642, 20.4%), diabetes (n = 412, 13.1%); asthma (n = 151, 4.8%), cancer (n = 263, 8.3%), and osteoarthritis (n = 205, 6.5%). Complete characteristics of participants are reported in the Table 1.

**Table 1:** Characteristics of participants involved in the assessment of the tool (n = 3152)

Characteristic	Raw sample (n = 3152)	Weighted sample (n = 3152)
Age, mean (SD)—yr	50.8 (15.0)	55.2 (16.9)
Female sex—no (%)	2209 (70.1)	1666 (52.9)
Educational level—no (%)		
Low	175 (5.6)	307 (9.7)
Middle school or equivalent	457 (14.5)	1776 (56.3)
High school or equivalent	227 (7.2)	436 (13.8)
Associate’s degree	644 (20.4)	277 (8.8)
Higher education	1649 (52.3)	356 (11.3)
Working status		
Working	1853 (58.7)	1319 (41.8)
Not working	1297 (41.1)	1831 (58.1)
Missing	2 (0.1)	2 (0.1)
Number of adults in household		
1	988 (31.3)	932 (29.6)
2	1751 (55.6)	1793 (56.9)
3	308 (9.8)	314 (10)
> 3	102 (3.2)	109 (3.5)
Missing	3 (0.1)	3 (0.1)
Number of children in household		
0	2421 (76.8)	2578 (81.8)
1	413 (13.1)	346 (11.0)
2	250 (7.9)	167 (5.3)
3	46 (1.5)	44 (1.4)
> 3	14 (0.4)	9 (0.3)
Missing	8 (0.3)	8 (0.3)
Lives with people ≥ 65 years old	598 (19.0)	830 (26.3)
Lives with other people having chronic conditions	822 (26.1)	808 (25.6)
Multimorbid—no (%)	1871 (59.4)	2001 (63.5)
Number of diseases, mean (SD)	2.61 (2.41)	2.76 (2.69)
Conditions—no (%)		
High blood pressure	517 (16.4)	642 (20.4)
Diabetes	302 (9.6)	412 (13.1)
Stroke or cardiac ischemic disease	41 (1.3)	56 (1.8)
Heart failure (other than ischemic diseases)	38 (1.2)	38 (1.2)
Asthma	198 (6.3)	151 (4.8)
COPD	84 (2.7)	120 (3.8)
Thyroid disease	187 (5.9)	133 (4.2)
Chronic kidney failure	86 (2.7)	84 (2.7)
Cancer	238 (7.6)	263 (8.3)
Osteoarthritis	195 (6.2)	205 (6.5)
Inflammatory rheumatic diseases	200 (6.3)	211 (6.7)

Weighted dataset was obtained by calibration on margins with weights for age, gender, and educational level derived from national census data describing the French population of patients with chronic conditions

Before consulting the tool, 1570 (49.8%) patients stated they would accept COVID-19 vaccination by any vaccine, 382 (12.1%) would accept at least one vaccine, 961 (30.5%) would refuse vaccination without more vaccine efficacy/safety data, and 239 (7.5%) would outright refuse vaccination. Patients’ perceptions of the importance of vaccination at the individual and population levels reflected their intention to receive vaccination but patients refusing to receive vaccination usually rated the importance of vaccination at population level higher than at individual level (Additional file 2).

### Outcomes

Among the patients who did not intend to be vaccinated at baseline (n = 1200 in the weighted data), 8.0% (n = 96 in the weighted data, Number needed to treat = 12.5) changed their mind after consulting the tool and reported that they would accept vaccination. These patients were mostly patients who indicated that they would refuse vaccination unless they had further data on vaccine efficacy and/or safety (97%, n = 93 in the weighted data). Patients’ characteristics significantly associated with a change of mind were (1) responding that they required further data on vaccine efficacy and/or safety (Odds Ratio 9.49, 95% confidence interval [CI] 3.00 to 29.96) and (2) high educational level (Odds Ratio 1.74, 95% CI 1.01 to 2.99) (Additional file 3**)**.

Patients who did not intend to be vaccinated at baseline found the tool moderately useful (mean ratings of 53.4 [SD = 1.8] and 35.6 [3.5] by patients asking for further data on vaccine efficacy and/or safety and by patients outright refusing vaccination, respectively). Among these patients, the tool slightly increased patients’ ratings of the importance of vaccination at individual level (mean increase of 4.0 [1.4] and 0.9 [0.4] points out of 100, for patients asking for further data on vaccine efficacy and/or safety and for patients outright refusing vaccination, respectively). However, the increase of their ratings of the importance of vaccination at population level was limited (mean increase of 2.3 [1.1] and 0.4 [1.2], respectively) (Additional file 4).

## Discussion

An interactive web tool presenting individualized information about the benefits and risks of COVID-19 vaccination moderately increased intention to be vaccinated among individuals with chronic conditions who initially intended to decline. As of the 28th of June 2021, about 33 million people in France (out of 64 million) received a first injection [15]. Yet, similar to other western countries, a glass ceiling begins to be reached and waiting lines are getting shorter in many vaccination centers. Persuading hesitant persons to receive vaccination is therefore of importance to achieve herd immunity.

Our tool was designed as a standalone, scalable, and effective method to address the need for transparent communication to help people make informed decisions based on reliable scientific data. The number needed to treat (i.e., the number of patients unsure or refusing to receive vaccination who, after consulting the tool, change their minds) was 12.5. Since the tool was released, more than 174,000 people have accessed it. The tool has been disseminated by the media in France and embedded on the official webpage of the French Government for COVID-19 information, since April 2021 [16]. It was enriched with data from Astra Zeneca’s and Johnson and Johnson’s vaccines and specific information regarding the risk of blood clots with low platelets have been added when users select the vaccine from Astra Zeneca [17–19].

Participants who changed their mind after consulting our tool were mainly those who asked for more information about the efficacy and safety of COVID-19 vaccination before seeing the tool. There were only 3 patients who outright refused vaccination changed their mind after consulting the tool. Similar to our results, other studies showed that outright vaccination refusal was strongly associated with a lower perceived severity of COVID-19 if infected, and that only a negligible proportion of those people would accept vaccination even if presented with an ideal vaccine in terms of efficacy and safety [3, 20]. This underlines that classic strategies to counter antivaccination attitudes (e.g., presenting data of the risks of not vaccinating) will not be effective in this population and that different strategies, such as incentivizing vaccination or complex combined strategies, should be considered [21].

Strengths of the study included the use of a before-and-after design, that was adapted for demonstrating the immediate impacts of the tool [22]. Indeed, as the before and after assessments were at the same time, it was unlikely that factors other than consulting the tool would have influenced patients’ intention to receive vaccination, thus limiting the biases inherent in this type of design. Second, we used statistical methods to enhance the representativeness of estimates and account for the sampling bias associated with the use of an online cohort of patients.

Our study was limited by several factors. First, the response rate was low, potentially owing to the very short recruitment period (7 days). Second, the tool was evaluated by itself, rather than during medical visits when clinicians could address patients’ questions and fears to enable shared decision-making in a person-centered approach. Our tool is only a necessary but not sufficient part of a shared decision-making intervention. Third, the tool required access to the Internet, which limited its impact on people with low literary or on those not comfortable engaging with online surveys. Fourth, our tool was evaluated before the recent concerns about a potential link between rare adverse effects and vaccination (e.g., blood clots and Adenovirus-based vaccines or myocarditis and mRNA vaccines) [5, 23]. Finally, our study highlighted that most patients who changed their minds were vaccine hesitant. For patients who outright refused vaccination, mistrust roots deeper than the lack of information on vaccine’s efficacy or benefits and is often connected with defiance towards the government or by deeper fears, unlikely to be modified by an evidence-based online intervention [24]. In this population, the use of decision aids during clinical encounters needs to be complemented by multi-faceted strategies based on restoring trust in healthcare providers and health authorities, as well as motivational interviewing during visits that best addresses patients’ values.

## Conclusion

We developed a simple and scalable interactive web tool on the benefits and risks of COVID-19 vaccination. For every 12.5 person unsure or refusing to receive vaccination, consulting our tool convinced one person to receive vaccination.

## Supplementary Information

**Additional file 1.** Data used to develop the tool.

**Additional file 2.** Patients’ perception of the importance of vaccination before consulting the tool.

**Additional file 3.** Demographic and clinical characteristics of patients changing their minds towards COVID-19 vaccination after consulting the tool. 3b: Association between participants’ characteristics and change of mind after consulting the tool.

**Additional file 4.** Patients’ perceptions of the tool’s usefulness and of the importance of vaccination at individual and population level after using the tool.

## Data Availability

All data collected for the study, including individual participant data and a data dictionary is available for research purposes, under the rules of the ComPaRe e-cohort described here: https://compare.aphp.fr. Please contact the corresponding author for further information.
