# Supplementary material for: Impact of an interactive web tool on patients’ intention to receive COVID-19 vaccination: a before-and-after impact study among patients with chronic conditions in France
Source: BMC Med Inform Decis Mak. 2021 Jul 31;21:228. doi: 10.1186/s12911-021-01594-8 (PMC8325218; doi:10.1186/s12911-021-01594-8)
Supplement: Supplementary file 1 — Additional file 1. Data used to develop the tool. [file 12911_2021_1594_MOESM1_ESM.docx]

# Supplemental material 1: Data used to develop the tool

Risk of death and hospitalization

Risk of death and hospitalization from COVID-19 were extracted from the publication of Salje et al, Science, 2020 [1]. In their article, the authors worked with daily hospitalization and death data from the SI-VIC database, maintained by the ANS (Agence du Numérique en Santé). The SI-VIC web portal was activated for the COVID-19 epidemic on 13 March 2020, with a progressive increase in the number of hospitals transmitting data. Data analyzed in the publication from Salje et al. include general ward (“Hospitalisation conventionnelle”) and ICU patients (“réanimation, soins intensifs or unité de surveillance continue”), and exclude patients hospitalized in psychiatric care (“Hospitalisation psychiatrique”), long-term care and rehabilitation care (“Soins de suite et réadaptation”) and emergency care patients (“Soins aux urgences”). Individuals whose only known status was deceased or discharged (3% of patients) were attributed a hospitalization date equal to the date of discharge or death.

Data were specifically extracted from Supplementary tables S1 and S2, available here: <https://science.sciencemag.org/content/suppl/2020/05/12/science.abc3517.DC1>.

Persistent symptoms at 2 months

Estimates of the risk of persistent symptoms of COVID-19 were extracted from the publication by Sudre et al. available on medRxiv [2] and available here:

<https://www.medrxiv.org/content/10.1101/2020.10.19.20214494v2>

In their papers, the authors used data from patient followed via the COVID 19 Symptom Study application which enabled the prospective recording of symptoms in the United Kingdom, the United States and Sweden (data collected since March 24, 2020). The study focused on 4,182 users who reported testing positive for CoV2-SARS by swab PCR with symptom onset between March 25, 2020 and June 30, 2020.

We used the risk of persistent symptoms at 56 days based on age and gender as presented in the Supplementary table 2 available here: <https://www.medrxiv.org/content/10.1101/2020.10.19.20214494v1.full-text>

Since the proportion of patients aged 18-30 years with persistent symptoms at 56 days was not reported in the article, we contacted the authors and used the provided unpublished values. Values for the proportion of patients aged 18-30 years with persistent symptoms at 56 days of 1.5% for women and 0.9% for men.

To relate the proportion of patients with persistent symptoms to a population of symptomatic patients (i.e. COVID-19 patients), we assumed that patients excluded because they did not report their characteristics, symptom onset date, symptom end date, did not have sufficient follow-up or did not log on to the application regularly had the same characteristics as the patients included (see Supplementary figure 1).

Vaccine Data

Estimates of vaccine efficacy/safety used the primary results of the trials evaluating vaccines. We assumed no heterogeneity of the treatment effect with age or gender. We also assumed that the treatment effect was similar in reducing risk of symptomatic disease (i.e. COVID-19), hospitalization, death and persisting symptoms.

Data on the efficacy and safety of the **Astra Zeneca vaccine** were extracted from [3]. We considered the vaccine efficacy to be 70.4% (30/5807 vs. 101/5829 patients reported COVID-19 after >14 days after the second dose of vaccine). This figure corresponded to the overall efficacy without taking into account the doses received. Some of the vaccine regimens tested had better efficacy. Regarding serious side effects, we reported serious side effects in the intervention arm that were considered possibly related to the experimental vaccine (n=2) with one case of transverse myelitis and fever above 40°C.

Data of the efficacy and safety of the **Pfizer BioNTech vaccine** were extracted from [4]. We considered the vaccine efficacy to be 95.027% (8/18198 vs. 162/18325 patients reported COVID-19 after >7 days after the second dose of the vaccine. Regarding serious side effects, we report serious side effects in the intervention arm and considered as possibly related to the experimental vaccine (n=4) with one vaccine-related shoulder injury, one right axillary lymphadenopathy, one paroxysmal ventricular arrhythmia and one paresthesia of the right leg.

Data of the efficacy and safety of the **Moderna vaccine** were extracted from [5]. We considered vaccine efficacy to be 94.1% (11 cases in the vaccine group [3.3 per 1000 person-years; 95% CI, 1.7 to 6.0] and 185 cases in the placebo group [56.5 per 1000 person-years; 95% CI, 48.7 to 65.3]) after >14 days after the second dose of vaccine. Regarding serious side effects, we reported serious side effects in the intervention arm that were considered possibly related to the experimental vaccine (n=8) (Autonomic nervous system imbalance, Dyspnea, Nausea, Vomiting, Rheumatoid arthritis, 2 Swelling face and Edema peripheral) (Supplementary table S15).

# References

1. Salje, H., et al., *Estimating the burden of SARS-CoV-2 in France.* Science, 2020. **369**(6500): p. 208-211.

2. Sudre, C.H., et al., *Attributes and predictors of Long-COVID: analysis of COVID cases and their symptoms collected by the Covid Symptoms Study App.* medRxiv, 2020: p. 2020.10.19.20214494.

3. Voysey, M., et al., *Safety and efficacy of the ChAdOx1 nCoV-19 vaccine (AZD1222) against SARS-CoV-2: an interim analysis of four randomised controlled trials in Brazil, South Africa, and the UK.* Lancet, 2020.

4. Polack, F.P., et al., *Safety and Efficacy of the BNT162b2 mRNA Covid-19 Vaccine.* N Engl J Med, 2020.

5. Baden, L.R., et al., *Efficacy and Safety of the mRNA-1273 SARS-CoV-2 Vaccine.* N Engl J Med, 2020.
