# Supplementary material for: Impact of an interactive web tool on patients’ intention to receive COVID-19 vaccination: a before-and-after impact study among patients with chronic conditions in France
Source: BMC Med Inform Decis Mak. 2021 Jul 31;21:228. doi: 10.1186/s12911-021-01594-8 (PMC8325218; doi:10.1186/s12911-021-01594-8)
Supplement: Supplementary file 2 — Additional file 2. Patients’ perception of the importance of vaccination before consulting the tool. [file 12911_2021_1594_MOESM2_ESM.docx]

# Supplemental material 2: Patients’ perception of the importance of vaccination before consulting the tool Patients’ perceptions of the importance of vaccination at individual and population level were assessed using rating scales ranging from 0 (not useful/important) to 100 (extremely useful / important)

| **Patients’ intent to receive COVID-19 vaccination before consulting the tool** | **Importance of vaccination at individual level**  **(before consulting the tool)** | **Importance of vaccination at population level**  **(before consulting the tool)** |
| --- | --- | --- |
| Yes, with any vaccine | 89.8 (0.7) | 91.6 (0.6) |
| Yes, but not with all vaccines | 82.7 (2.1) | 82.7 (2.3) |
| No, I prefer to wait for more vaccine efficacy/safety data | 38.3 (1.5) | 54.5 (1.5) |
| No, I don’t want to be vaccinated at all | 8.9 (2.0) | 25.5 (2.9) |
