# Supplementary material for: Impact of an interactive web tool on patients’ intention to receive COVID-19 vaccination: a before-and-after impact study among patients with chronic conditions in France
Source: BMC Med Inform Decis Mak. 2021 Jul 31;21:228. doi: 10.1186/s12911-021-01594-8 (PMC8325218; doi:10.1186/s12911-021-01594-8)
Supplement: Supplementary file 3 — Additional file 3. Demographic and clinical characteristics of patients changing their minds towards COVID-19 vaccination after consulting the tool. 3b: Association between participants’ characteristics and change of mind after consulting the tool. [file 12911_2021_1594_MOESM3_ESM.docx]

# Supplemental material 3: Demographic and clinical characteristics of patients changing their minds towards COVID-19 vaccination after consulting the tool. Weighted dataset was obtained by calibration on margins with weights for age, gender, and educational level derived from national census data describing the French population of patients with chronic conditions

| **Characteristic** | **Did not change their mind after consulting the tool**  **(n=1104)** | **Changed their mind after consulting the tool**  **(n=96)** |
| --- | --- | --- |
| Age, mean (SD) – yr | 50 (17) | 52 (14) |
| Female sex – no (%) | 758 (69) | 68 (71) |
| Educational level – no (%)  Low  Middle school or equivalent  High school or equivalent  Associate’s degree  Higher education | 130 (12)  676 (61)  140 (13)  82 (7)  77 (7) | 11 (11)  48 (51)  16 (17)  10 (10)  10 (11) |
| Working status  Working  Not working  Missing | 418 (38)  781 (65)  1 (0) | 54 (57)  42 (43)  0 (0) |
| Number of adults in household ≥ 1 | 378 (34) | 23 (24) |
| Number of children in household ≥ 1 | 867 (78) | 73 (76) |
| Lives with people ≥ 65 years old | 511 (46) | 55 (58) |
| Lives with other people having chronic conditions | 461 (42) | 50 (52) |
| Conditions – no (%)  High blood pressure  Diabetes  Stroke or cardiac ischemic disease  Heart failure (other than ischemic diseases)  Asthma  COPD  Thyroid disease  Chronic kidney failure  Cancer  Osteoarthritis  Inflammatory rheumatic diseases | 169 (15)  127 (11)  4 (0)  5 (0)  45 (4)  42 (4)  38 (3)  27 (2)  60 (5)  85 (8)  61 (6) | 10 (10)  9 (10)  0 (0)  4 (4)  0 (0)  1 (1)  3 (3)  1 (1)  13 (13)  3 (3)  11 (12) |

**Supplemental material 3b. Association between participants’ characteristics and change of mind after consulting the tool**. We performed a logistic regression analysis of complete cases, accounting for weights obtained after calibration on margins for sex, age categories and educational level by using data from a national census describing the French population self-reporting at least one chronic condition.

| **Characteristic** | **Odds Ratio**  **(95% Confidence Interval)** |
| --- | --- |
| Sex | 1.59 (0.62 to 4.04) |
| Age | 1.02 (0.99 to 1.04) |
| High educational level | 1.74 (1.01 to 2.99) |
| household with > 1 adult (including the patient) | 1.99 (0.90 to 4.40) |
| household with ≥ 1 children | 1.08 (0.45 to 2.59) |
| living with a person over 65 years old | 0.82 (0.34 to 1.99) |
| living with a person having a chronic condition | 0.71 (0.32 to 1.56) |
| Refusing vaccination until further data on vaccine efficacy and/or safety is obtained | 9.49 (3.00 to 29.96) |
