# Supplementary material for: Impact of an interactive web tool on patients’ intention to receive COVID-19 vaccination: a before-and-after impact study among patients with chronic conditions in France
Source: BMC Med Inform Decis Mak. 2021 Jul 31;21:228. doi: 10.1186/s12911-021-01594-8 (PMC8325218; doi:10.1186/s12911-021-01594-8)
Supplement: Supplementary file 4 — Additional file 4. Patients’ perceptions of the tool’s usefulness and of the importance of vaccination at individual and population level after using the tool. [file 12911_2021_1594_MOESM4_ESM.docx]

# Supplemental material 4: Patients’ perceptions of the tool’s usefulness and of the importance of vaccination at individual and population level after using the tool. Rating scales ranged from 0 (not useful/important) to 100 (extremely useful / important)

| **Patients’ intent to receive COVID-19 vaccination before consulting the tool** | **Rating of the tool’s usefulness** | **Importance of vaccination at individual level**  **(after consulting the tool)** | **Importance of vaccination at population level**  **(after consulting the tool)** |
| --- | --- | --- | --- |
| Yes, with any vaccine | 73.9 (1.2) | 91.6 (0.7) | 92.9 (0.5) |
| Yes, but not with all vaccines | 67.5 (2.7) | 85.2 (1.9) | 84.3 (2.3) |
| No, I prefer to wait for more vaccine efficacy/safety data | 53.4 (1.8) | 42.3 (1.8) | 56.8 (1.8) |
| No, I don’t want to be vaccinated at all | 35.6 (3.5) | 9.8 (2.0) | 25.9 (2.7) |
